# Supplementary material for: Unique transcriptional and protein-expression signature in human lung tissue-resident NK cells
Source: Nat Commun. 2019 Aug 26;10:3841. doi: 10.1038/s41467-019-11632-9 (PMC6710242; doi:10.1038/s41467-019-11632-9)
Supplement: Supplementary file 1 — Supplementary figures [file 41467_2019_11632_MOESM1_ESM.pdf]

Supplementary figures

Unique transcriptional and protein-expression signature in human lung tissue-resident NK cells

Marquardt *et al*.

Supplementary Figure 1

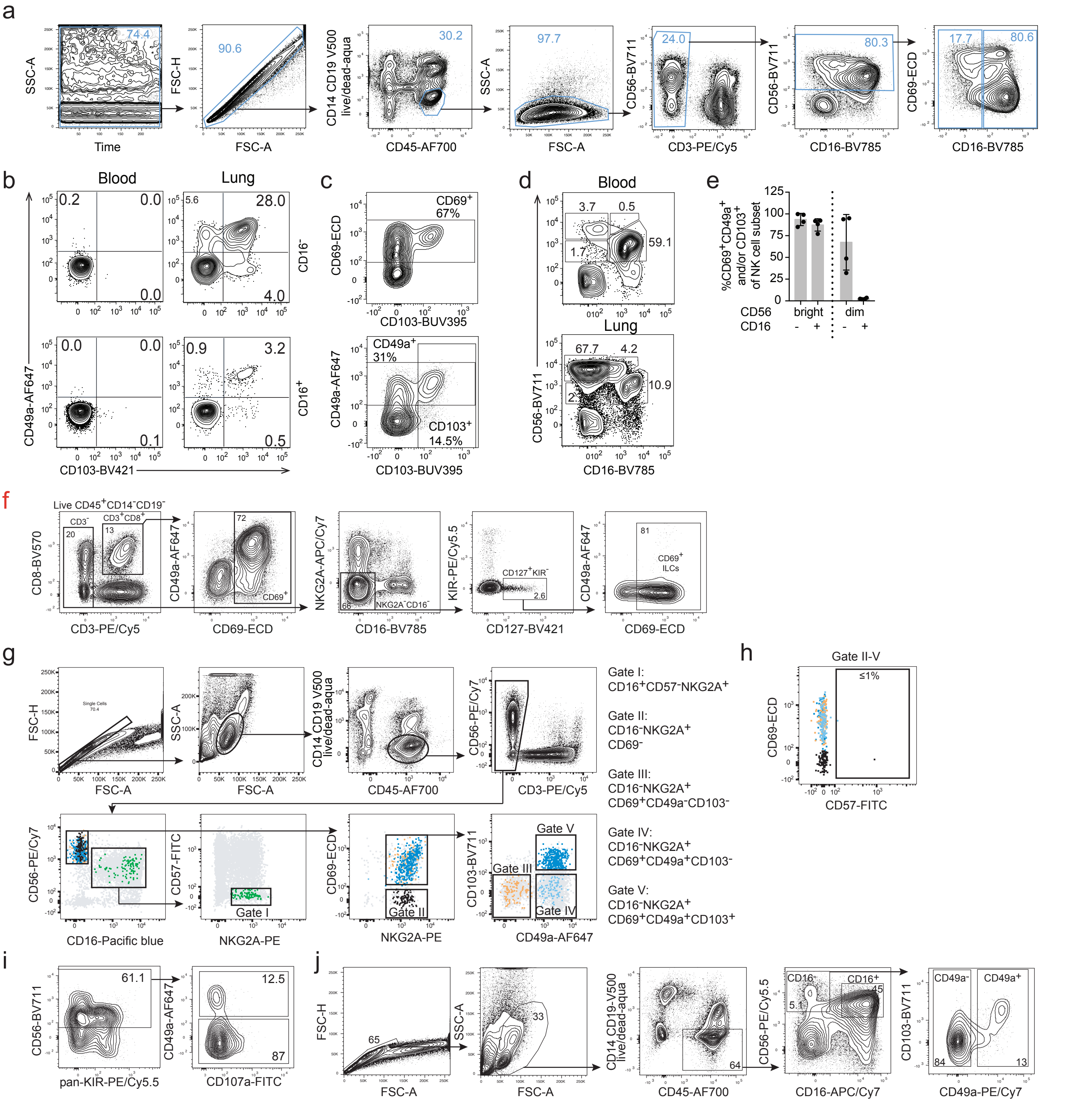

Supplementary Figure 1: Identification of NK cell subsets in human lung. **(a)** Gating strategy to identify NK cells in human lung tissue (related to Fig. 1a,b, and 2a-h). (**b**) Representative contour plots showing expression of CD49a and CD103 on CD16- and CD16+ NK cells in blood and lung respectively. **(c)** Representative contour plots showing expression ofCD69, CD49a, and CD103 for setting boolean gate [CD69+ AND (CD49a+ OR CD103+)]. The boolean gate thus includes CD69+CD49a+CD103+, CD69+CD49a+CD103-, and CD69+CD49a-CD103+ NK cells, and is referred to as "CD69+CD49a+ and/or CD103+" (related to Figs. 1b-f and 2a-h). (**d**) Representative contour plots for identifying different NK cell subsets based on CD56 and CD16 among CD45+CD3-CD14-CD19- live lymphocytes. Frequencies of the respective subsets are indicated in the plots. (**e**) Summary of frequencies of CD69+CD49a+ and/or CD103+ cells among CD56brightCD16-, CD56brightCD16+, CD56dimCD16-, and CD56dimCD16+ NK cells in donors with high frequencies of CD16+CD69+CD49a+CD103+ NK cells (identified in Fig. 1b) (n=4). (**f**) Gating strategy to identify CD69+ ILCs and CD8+ T cells (related to Fig. 1h, i). (**g**) Gating strategy for sort for RNA sequencing analysis (related to Fig. 2). (**h**) CD57 expression on cells in gate II-V. Note that ≤1% of cells express CD57, and thus all sorted subsets effectively are NKG2A+CD57-. (**i**) Gating strategy to identify CD49a+ and CD49a- NK cells. Left plot shows CD45+CD3-CD14-CD19- live cells gated as in Fig. S1a. (related to Fig. 4b, c). (**j**) Gating strategy to sort CD16+ and CD16- NK cells (related to Fig. 4d), as well as CD49a+CD16- and CD49a-CD16- NK cells (related to to Fig. 4e-g).
Source Data are provided as Source Data file.

Supplementary Figure 2

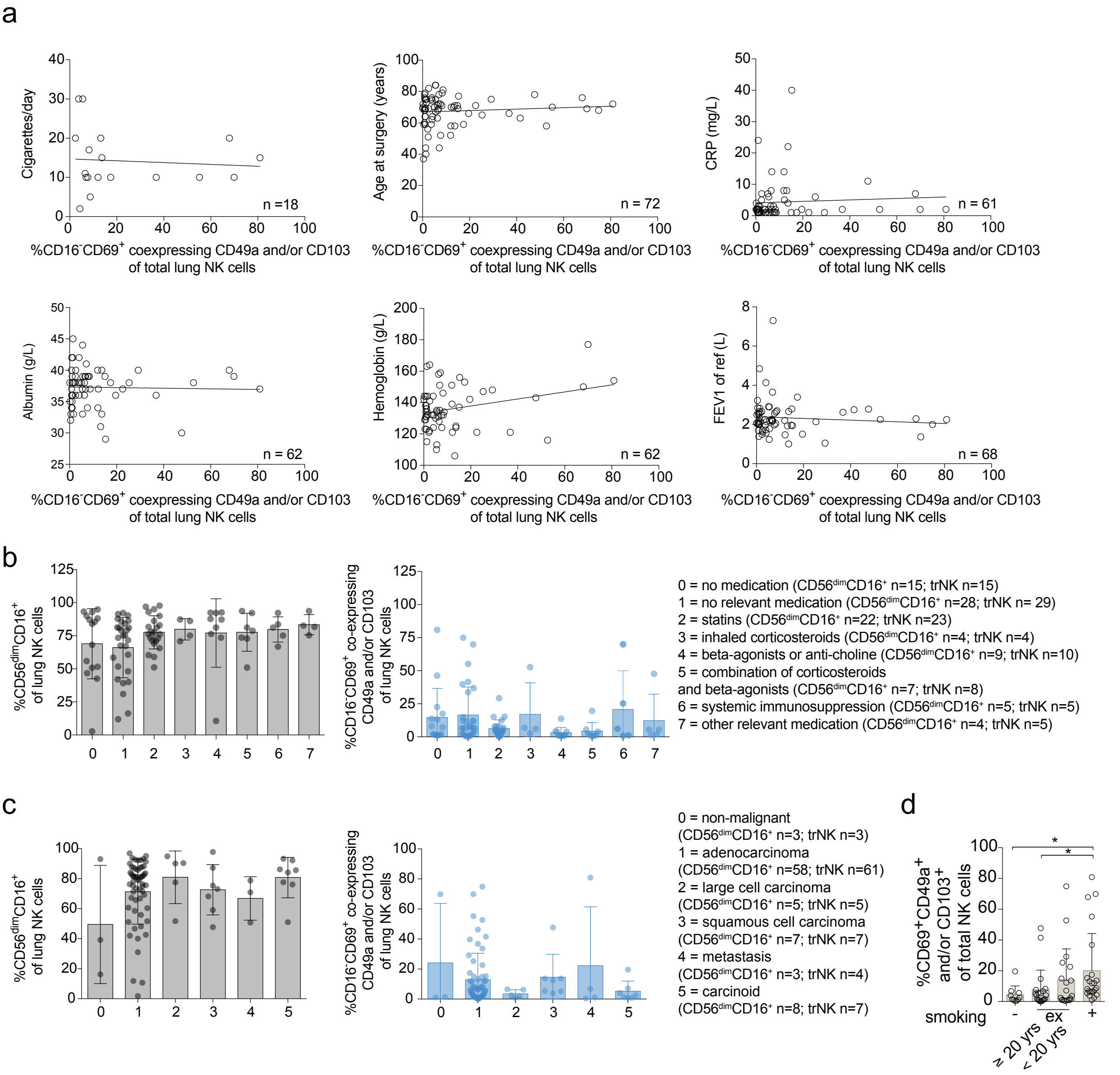

Supplementary Figure 2: Frequencies of human lung NK cell subsets are independent from clinical parameters. **(a)** Linear regression analysis of frequencies of CD16$^-$CD69$^+$ NK cells co-expressing CD49a and/or CD103 in lung in correlation to different patient characteristics. **(b)** Frequencies of CD56^{dim}CD16$^+$ (left panel) and CD16$^-$CD69$^+$ NK cells co-expressing CD49a and/or CD103 (right panel) in lung were analyzed, and patients were grouped according to the medication (indicated in numbers). **(c)** Frequencies of CD56^{dim}CD16$^+$ (left panel) and trNK cells (CD16$^-$CD69$^+$ NK cells co-expressing CD49a and/or CD103; right panel) in lung were analyzed, and patients were grouped according to the type of cancer (indicated in numbers). (**d**) Frequencies of CD69$^+$CD49a$^+$ and/or CD103$^+$ NK cells among total NK cells in lungs from non-smokers (-) (n=10), ex-smokers with smoking cessation more (n=21) or less than 20 years (n=19) before surgery (ex), and active smokers (+) (n=23). Mean ± SD is shown. Mann-Whitney test, *p<0.05, **p<0.01.
Source data are provided as Source Data file.

Supplementary Figure 3

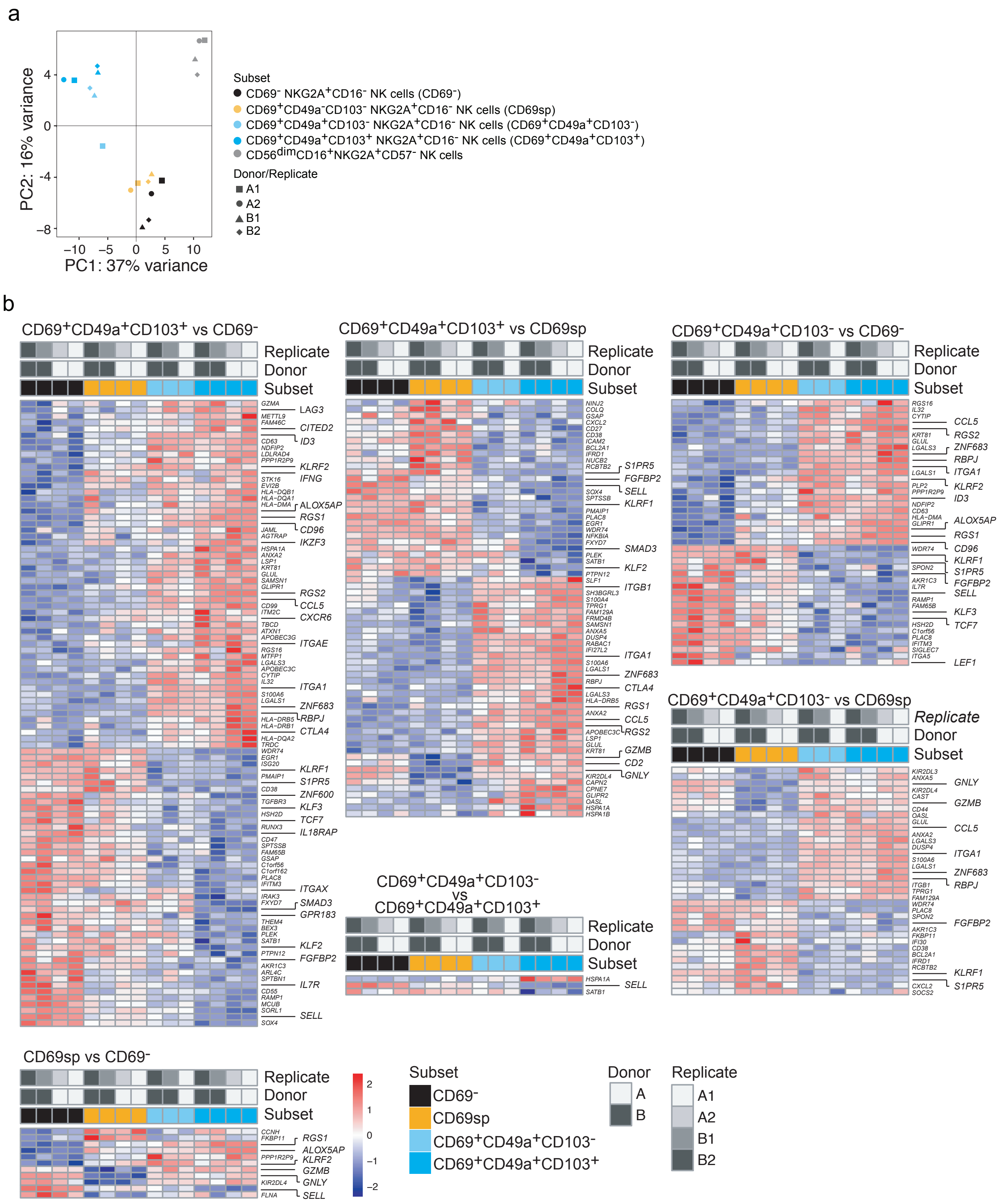

Supplementary Figure 3: Distinct gene expression patterns in NK cell subsets in human lung. **(a)** Principal component analysis of CD69⁻, CD69⁺CD49a⁻CD103⁻ (CD69sp), CD69⁺CD49a⁺CD103⁻, CD69⁺CD49a⁺CD103⁺ NKG2A⁺CD16⁻ NK cells, and CD56dimCD16⁺NKG2A⁺CD57⁻ NK cells in human lung. **(b)** Heatmaps depicting differentially expressed genes between individual subsets of NKG2A⁺CD16⁻ NK cells in human lung (padj<0.01, log2FC>1). Color scale depicts z-score.

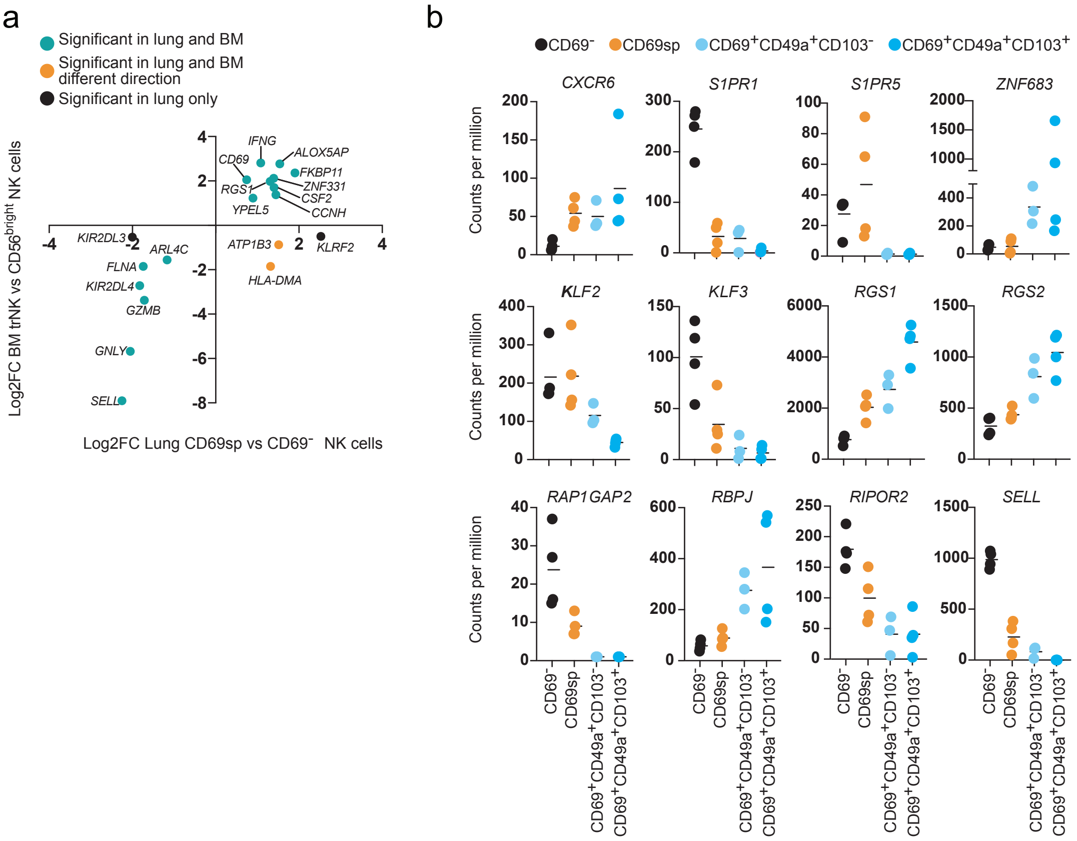

Supplementary Figure 4: CD69sp in lung and trNK cells in bone marrow share expression patterns of some genes. **(a)** Log2-fold change (Log2FC) in gene expression between CD69$^+$CD49a$^-$CD103$^-$ (CD69sp) and CD69$^-$ NKG2A$^+$CD16$^-$ NK cells in lung versus Log2FC in gene expression between trNK cells and CD56^{bright} NK cells in bone marrow (BM). Data for NK cells in BM are from Melsen *et al* [20].
(b) Gene expression levels (counts per million reads) for selected genes associated with tissue-residency are shown for CD69$^-$, CD69$^+$CD49a$^-$CD103$^-$ (CD69sp), CD69$^+$CD49a$^+$CD103$^-$, and CD69$^+$CD49a$^+$CD103$^+$ NKG2A$^+$CD16$^-$ NK cells in human lung.

Supplementary Figure 5

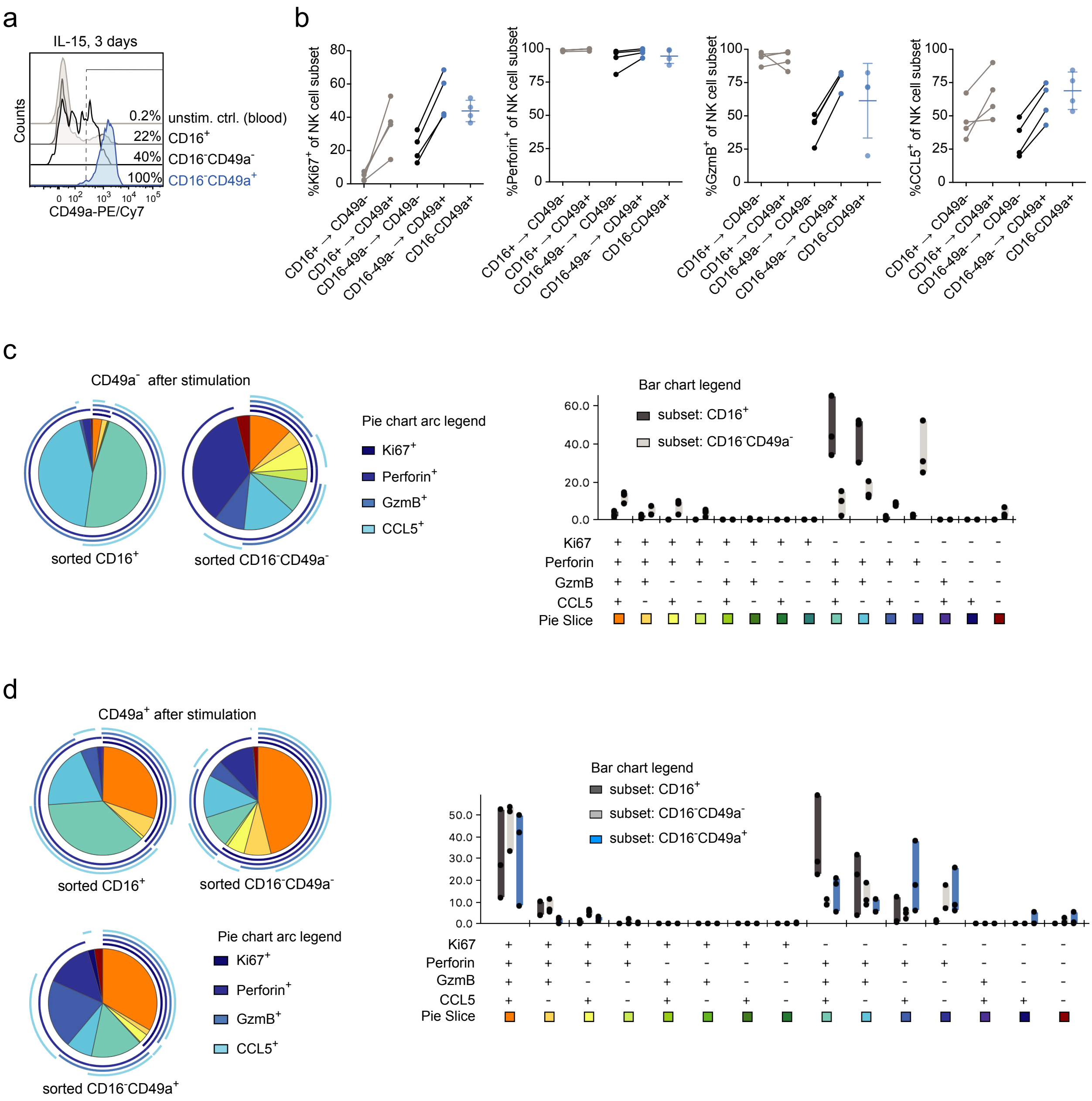

Supplementary Figure 5: De novo IL-15-induced CD49a+ NK cells derived from CD49a- lung NK cells are polyfunctional. **(a)** Representative overlay of CD49a expression on sorted lung NK cell subsets following stimulation with IL-15 for 3 days. Unstimulated peripheral blood NK cells served as control for expression of CD49a. Numbers indicate CD49a+ NK cells in the respective gate as depicted in the overlay. **(b)** Summary of data for expression of Ki67, perforin, granzyme B (GzmB), and CCL5 on sorted NK cell subsets that did or did not upregulate CD49a following stimulation with IL-15 (n=4). **(c)** SPICE analysis of expression of Ki67, perforin, granzyme B (GzmB), and CCL5 of sorted NK cell subsets that remained CD49a- or **(d)** upregulated CD49a following stimulation with IL-15 for 3 days. **(c, d)** Floating bars min to max, (n=3). Source data are provided as a Source Data file.
